# Tolerability of inhaled N-chlorotaurine in the pig model

**DOI:** 10.1186/1471-2466-9-33

**Published:** 2009-07-14

**Authors:** Ralf Geiger, Benedikt Treml, Anna Pinna, Linn Barnickel, Harald Prossliner, Hannes Reinstadler, Michael Pilch, Maria Hauer, Christoph Walther, Hans-Jörg Steiner, Thomas Giese, Andreas Wemhöner, Sabine Scholl-Bürgi, Waldemar Gottardi, Roland Arnitz, Consolato Sergi, Markus Nagl, Alexander Löckinger

**Affiliations:** 1 Department of Pediatrics, Division of Cardiology, Pulmology, Allergology and Cystic Fibrosis, Innsbruck Medical University, Innsbruck, Austria; 2 Division of Neonatology, Neuropediatrics and Inborn errors of metabolism, Innsbruck Medical University, Innsbruck, Austria; 3 Department of Anaesthesiology and Critical Care Medicine, Innsbruck Medical University, Innsbruck, Austria; 4 Department of Hygiene, Microbiology and Social Medicine, Division of Hygiene and Medical Microbiology, Innsbruck Medical University, Innsbruck, Austria; 5 Institute of Pathology, Innsbruck Medical University, Innsbruck, Austria; 6 Department of Immunology, University of Heidelberg, Heidelberg, Germany; 7 Technical University Dresden, University Hospital Dresden, Department for Pediatric Intensive Care and Neonatology, Dresden, Germany

## Abstract

**Background:**

N-chlorotaurine, a long-lived oxidant produced by human leukocytes, can be applied in human medicine as an endogenous antiseptic. Its antimicrobial activity can be enhanced by ammonium chloride. This study was designed to evaluate the tolerability of inhaled N-chlorotaurine (NCT) in the pig model.

**Methods:**

Anesthetized pigs inhaled test solutions of 1% (55 mM) NCT (n = 7), 5% NCT (n = 6), or 1% NCT plus 1% ammonium chloride (NH_4_Cl) (n = 6), and 0.9% saline solution as a control (n = 7), respectively. Applications with 5 ml each were performed hourly within four hours. Lung function, haemodynamics, and pharmacokinetics were monitored. Bronchial lavage samples for captive bubble surfactometry and lung samples for histology and electron microscopy were removed.

**Results:**

Arterial pressure of oxygen (PaO_2_) decreased significantly over the observation period of 4 hours in all animals. Compared to saline, 1% NCT + 1% NH_4_Cl led to significantly lower PaO_2 _values at the endpoint after 4 hours (62 ± 9.6 mmHg vs. 76 ± 9.2 mmHg, p = 0.014) with a corresponding increase in alveolo-arterial difference of oxygen partial pressure (AaDO_2_) (p = 0.004). Interestingly, AaDO_2 _was lowest with 1% NCT, even lower than with saline (p = 0.016). The increase of pulmonary artery pressure (PAP) over the observation period was smallest with 1% NCT without difference to controls (p = 0.91), and higher with 5% NCT (p = 0.02), and NCT + NH_4_Cl (p = 0.05).

Histological and ultrastructural investigations revealed no differences between the test and control groups. The surfactant function remained intact. There was no systemic resorption of NCT detectable, and its local inactivation took place within 30 min. The concentration of NCT tolerated by A549 lung epithelial cells *in vitro *was similar to that known from other body cells (0.25–0.5 mM).

**Conclusion:**

The endogenous antiseptic NCT was well tolerated at a concentration of 1% upon inhalation in the pig model. Addition of ammonium chloride in high concentration provokes a statistically significant impact on blood oxygenation.

## Background

N-chlorotaurine (Cl-HN-CH_2_-CH_2_-SO_3_^-^), the N-chloro derivative of the amino acid taurine, is a long-lived oxidant produced by activated human granulocytes and monocytes [1], and in low concentration probably also in macrophages [2]. It is the main representative of chloramines, i.e. oxidizing R-NHCl compounds, created during the oxidative burst [3]. Its main functions in vivo are thought to be detoxification of HOCl by its formation (reaction of HOCl with taurine) [1,3] and termination of inflammation by downregulation of proinflammatory cytokines and interleukins [4,5], but also activation of white blood cells to eradicate pathogens is conceivable (Giese T, Meuer S, Heidelberg, personal communication).

Eventually, the synthesis of the pure crystalline sodium salt of N-chlorotaurine (NCT, molecular weight 181.57 g/mol) in our laboratory succeeded [6]. A 90% stability of both the pure product and the aqueous solution at 2–4°C per year makes it suitable for practical use [6]. This facilitated a comprehensive investigation of its antimicrobial properties. Studies disclosed broad spectrum bactericidal (Gram-positive and Gram-negative bacteria), fungicidal (yeasts and moulds) and virucidal activity of NCT [7-10] without development of resistance [7].

The activity of NCT against bacteria and fungi could be significantly enhanced by addition of ammonium chloride (NH_4_Cl) [7,10]. This phenomenon is caused by chlorine transfer from NCT to ammonium ("transhalogenation") at which the lipophilic and therefore stronger microbicidal monochloramine (NH_2_Cl) is formed [3,7]. As a consequence, the activity of NCT against bacteria and particularly fungi is higher in inflammatory exudates than in buffer solution since the exudates contain ammonium [10].

The antimicrobial and immune-modulatory features encouraged investigations of the usability of 1% (55 mM) NCT solution as a topical antiseptic for the treatment of infections in human medicine. Promising indications proved to be conjunctivitis [11,12], purulent crural ulcerations[13], and otitis externa [14]. In a rabbit viral conjunctivitis model some efficacy of NCT and superior efficacy of NCT plus ammonium chloride was found [11]. In mouse models, NCT was beneficial in experimentally induced arthritis [15,16].

For deep airway infections, inhalative therapy with antibiotics is performed in some special cases (e.g. Pneumocystis jirovecii and cystic fibrosis) [17], but may also be beneficial in further indications [18]. Therapeutic and prophylactic inhalative use of a well-tolerated antiseptic in bronchopulmonary infections may be an interesting perspective. NCT as an endogenous compound anyway present in small amounts at inflammatory sites including the bronchi [19] may meet these requirements. Its formation has been shown to detoxify hypochlorous acid (taurine + HOCl → NCT + H_2_O) and to protect lung epithelial cells by this mechanism [20]. The ciliary beat frequency, a very sensitive parameter for testing toxicity, of mucosa samples gained from the nasal cavity upon conchotomy was only minimally and reversibly influenced by 20 min in 1% NCT, while an anaesthetic solution used in the everyday otorhinolaryngological practice caused a marked and irreversible reduction [21].

Recently, a pig model at our university has been established for investigation of the acute respiratory distress syndrome [22-24]. This model can be used for testing substances applied as aerosol via an inhalation device. Therapeutic effects can be detected within short time in this model [22,25], and it was found suitable for our investigations.

The aim of this study was to investigate the tolerability of inhaled NCT in our pig model. In addition, the combination of NCT and ammonium chloride was tested.

## Methods

### Test solutions

Pure NCT as a crystalline sodium salt (molecular weight 181,57) was prepared according to [6]. Purity was proved by spectrophotometry [6]. Ammonium chloride at the highest available purity was purchased from Merck (Darmstadt, Germany). Reagents were dissolved in sterile and pyrogen-free distilled water to solutions with a final concentration of 1% NCT (55 mM), 5% NCT (275 mM) as well as 1% NCT plus 1% NH_4_Cl (187 mM). Solutions were prepared as required, sterile-filtered and stored at 2–4°C for maximally one week. To warrant double-blinding they were filled in similar flasks than sterile and pyrogen-free saline which served as a control. All flasks were numbered consecutively in accordance with the randomization code.

### Animals

The experiment was approved by the Austrian Federal Animal Investigational Committee, and animals were managed in accordance with the National Institutes of Health guidelines . Healthy, 4-week-old white farm pigs were used in the study. They were anesthetized during the whole experiment.

### Study design and treatment

The pigs were randomly assigned to one of the three test groups (1% NCT, 5% NCT, 1% NCT plus 1% NH_4_Cl) or to the control group (0.9% saline). Six animals per group were included plus 1 animal per group as a reserve. Maximally 2 pigs were treated and evaluated on the same day. Application of test solutions and measurements of evaluation parameters were performed in a blinded way. Thirty min after anaesthesia (see below), baseline parameters were recorded. Again 30 min later, the first inhalation was performed, followed by three further inhalations at intervals of 1 hour with 5 ml of one of the test substances or saline. One hour after the last inhalation the animals were euthanized, and lung samples were removed. Parameters of oxygenation and circulation were monitored. Baseline values and values every 30 min after the first inhalation were recorded. Blood samples were also taken at the baseline, 2 h later, and 30 min after the last inhalation.

### Anaesthesia of pigs

Anaesthesia was induced with ketamine (50 mg/kg IM) and atropine (0.01 mg/kg IM), followed by intravenous propofol (2 to 4 mg/kg). After the trachea had been intubated, lungs were ventilated in volume controlled mode (Evita 4, Dräger) at an inspiratory fraction of oxygen (FIO_2_) of 0.4 and a tidal volume (VT) of 10 mL/kg at 20 breaths/minute, positive end expiratory pressure set at 5 cm H_2_O. Tidal volume was then adjusted to achieve an arterial pressure of carbon dioxide (PaCO_2_) between 35 and 40 mm Hg, resulting in a minute ventilation of 150 to 170 mL/kg·min^-1^. Anaesthesia was maintained with propofol (10 to 15 mg/kg/h) and piritramide boluses (15 mg each). Ringer's solution (6 mL/kg/h) and a 3% gelatine solution (4 mL/kg/h) were administered throughout the procedure. A standard lead II ECG was used to monitor cardiac rhythm. Body temperature was maintained between 38°C and 39°C by using an electric heating blanket.

### Animal instrumentation

Venous catheters were inserted percutaneously into auricular veins for inert gas infusion and continuous infusion of propofol. A no. 7F thermistor-tipped Swan-Ganz catheter (Baxter Edwards, Irvine, CA) was inserted into the right jugular vein and advanced into a main pulmonary artery by use of direct-pressure monitoring. This allowed measurement of cardiac output, PAPM, and mixed venous blood sampling. The left femoral artery was cannulated for measurement of systemic pressure and arterial blood gas sampling.

### Haemodynamic measurements

Mean arterial blood pressure (MAP), central venous pressure (CVP), and pulmonary artery occlusion pressure (PCWP) were measured with an ICU monitor (Servomed; Hellige GmbH, Frieburg, Germany) with standard pressure transducers (model 1290A; Hewlett-Packard, Böblingen, Germany) that had been zeroed to the level of the right atrium. Cardiac output was measured with a Baxter-Vigilance Monitor (Edwards Critical Care Division, Irvine, CA) and a no. 7F Swan-Ganz catheter. The mean of four determinations of cardiac output was recorded. Pulmonary and systemic vascular resistance (PVRI, SVRI) was calculated using standard formulas. Normalized values were calculated by using a converting factor of 0.0947·kg^2/3 ^[26].

### Blood Gas and Metabolic Measurements

Arterial and mixed venous samples (2 ml each) were collected simultaneously and immediately analyzed for oxygen and carbon dioxide partial pressure, pH, haemoglobin concentration, and haematocrit.

### Respiratory Measurements

Airway pressures, expiratory tidal volume, expiratory minute volume, and respiratory rate values were recorded by the built-in detectors of the ventilator. Ventilation-perfusion distributions were determined using the multiple inert gas elimination technique (MIGET) [27,28].

### Protocol

After instrumentation of the animals and a stabilization phase of 30 minutes, baseline measurements (haemodynamics, blood gases) were taken. Each set of measurements included heart rate, MAP, and CVP, mean pulmonary arterial pressure (PAPM), and PCWP. Inert gas as well as arterial and mixed venous blood gas samples were taken at the same time.

Inhalation was carried out by an ultrasonic nebulizer (OPTINEB^®^-ir Modell/Type: ON-100/2–2.4 MHz from NEBU-TEC med. Produkte Eike Kern GmbH, Elsenfeld, Germany). Delivery of the aerosol was provided during the inspiration phase by side port connection to the inspiratory side of the Y-piece. At the end of the experimental runs, animals were killed using pentobarbital.

### Pharmacokinetics of NCT

To address systemic uptake of NCT, the taurine concentration in the serum was determined at the baseline, after 2 inhalations and 30 min after the last inhalation. If NCT in amounts used for inhalation comes into contact with blood, it is immediately inactivated by thio groups and degrades into taurine and chloride. Therefore, taurine is an ideal measure for systemic uptake of topically applied NCT. Taurine was analyzed by ion-exchange chromatography with ninhydrin detection (AminoTac, JLC-500/V, Jeol) [29]. Since the machine automatically provides the results, we evaluated 28 further amino acids in total.

To address the local pharmacokinetics in the lung, immediately after euthanizing the animals, the chest was opened and a peripheral bronchus was irrigated with 10 ml of saline. Potassium iodide was added in excess, and the formed coloured tri-iodide was measured spectrophotometrically at 350 nm, its maximum wavelength (absorption coefficient 22900/mol/cm [1]).

### Histology

Subsequent to euthanizing the animal, artificial ventilation was continued up to removal of lung samples of the upper and lower lobes. Lung tissue was subjected to histological processing according to standard procedures as follows: (i) formalin fixation and paraffin-embedding; (ii) freezing with isopentane-N2, and storage at -70°C: (iii) fixation with glutaraldehyde (2%). Moreover, fresh tissue was used to extract protein, DNA and RNA according to commercial kits instructions. The tissue was routinely stained with haematoxylin and eosin, periodic acid Schiff (glycogen staining), elastica van Gieson (elastic fibers of the intrapulmonary blood vessels), and reticulin (reticulin fibers). The haematoxylin-eosin staining was used to evaluate the infiltration with neutrophilic granulocytes, eosinophilic granulocytes, lymphocytes, erythrocytes, atelectasis, bronchiolitis, bronchitis, lymphangiectasia, and capillary congestion. The PAS staining was used to evaluate the variability of the amount of goblet cells, mucus, detachment of epithelial cells and epithelial changes. The elastica van Gieson was used to detect the fragmentation of the external and internal laminae of the blood vessels. The reticulin staining was used to detect collapse of fibers, thinning or thickening of the alveolar septa, the presence of emphysema, bullae and thickening of the pleura.

### Electron Microscopy

Transmission electron microscopy (TEM) was used to evaluate possible effects of NCT to subcellular constituents (organelles, endomembranes, cytoskeleton). Small pieces from swine lung (approximately 1 mm in diameter) were collected immediately after slaughtering and fixed in 1% phosphate buffered glutaraldehyde for at least 24 hours. After post fixation in a 1% aqueous osmium solution the probes were dehydrated in a graded series of ethyl alcohol (30, 50, 70, 90, 3 × 100%) and finally embedded in epoxy resin (Fluka, Germany).

Thin sections were prepared with a Reichert Jung Ultracut E (Leica Instruments, UK) mounted on formvar coated copper grids (Polysciences, USA) and counterstained with uranyl acetate and lead citrate. Ultrastructural investigations were performed with a Zeiss EM 109 (Zeiss, GER). Micrographs were taken on ILFORD PAN F 50 negative film and digitalized with a Nikon ED 9000 film scanner (Nikon, JPN). Since manual measurement can be error-prone, we used an operator-interactive, semi-automated method using ImageJ , an open-source platform running on Windows^® ^environment [30].

### In situ determination of cytokines

Expression of interleukins (IL) IL-1, IL-8, TNF-α, and haem oxygenase 1 was investigated by mRNA analysis according to [31].

#### Real-Time RT-PCR

Biopsies were collected in RNAlater (Ambion, Austin, TX, USA), and stored at -20°C until analysis. Tissue was disrupted by 3 to 4 runs with the RiboLyser (ThermoHYBAID, Heidelberg) in lysing matrix „D” tubes (Q-BIOgen, Heidelberg) containing 400 μl lysis buffer from the MagnaPure mRNA Isolation Kit II (ROCHE Diagnostics, Mannheim). 300 μl of the lysate was collected and mixed with 600 μl capture buffer containing oligo-dT. After centrifugation at 13000 rpm for 5 min, 880 μl of this mix was transferred into a MagnaPure sample cartridge and mRNA was isolated with the MagnaPure-LC device using the mRNA-II standard protocol. The elution volume was set to 50 μl.

An aliquot of 8.2 μl mRNA was reversely transcribed using AMV-RT and oligo-(dT) as primer (First Strand cDNA synthesis kit, Roche) according to the manufactures protocol in a thermocycler. After termination of the cDNA synthesis, the reaction mix was diluted to a final volume of 500 μl and stored at -20°C until PCR analysis. Primer sets for pig GAPDH, IL-1b, IL-8, HO-1 and TNF-alpha optimized for the LightCycler^® ^(RAS, Mannheim Germany) were developed and purchased from SEARCH-LC GmbH, Heidelberg. The PCR was performed with the LightCycler^® ^FastStart DNA Sybr GreenI kit (RAS) according to the protocol provided in the parameter specific kits. To control for specificity of the amplification products, a melting curve analysis was performed. The copy number was calculated from a standard curve, obtained by plotting known input concentrations of four different plasmids at log dilutions to the PCR-cycle number (CP) at which the detected fluorescence intensity reaches a fixed value. This approach dramatically reduced variations due to handling errors over several logarithmic dilution steps. To correct for differences in the content of mRNA, the calculated copy numbers were normalized according to the average expression of the housekeeping gene GAPDH. Values were thus given as input adjusted copy number per μl of cDNA.

### Influence of the inhalations on the surfactant

Captive bubble surfactometry was performed to evaluate the function of the surfactant. Immediately after euthanasia, a bronchus was irrigated with 10 ml saline, and the lavage fluid was collected (bronchiolo-alveolar lavage, BAL). BAL samples were collected, spun and kept frozen at -20°C before use. Because of the high efforts for this method, only a total of six samples could be investigated, two from the NCT + NH_4_Cl, two from the saline, and one each from the 1% NCT and 5% NCT group. As an internal quality control, three measurements using porcine surfactant (Curosurf^®^, 80 mg/ml stock solution) were performed.

The phospholipid concentration was determined, and all samples and controls were diluted with TRIS-buffer (Sigma) to a concentration of 1 mg/ml lecithin. 70 μl Curosurf^R ^had to be diluted with 3930 μl buffer. Measurements of quasi-static surface tension measurements of BAL were performed with captive bubble surfactometry [32]. For film formation a 5-min adsorption period followed the introduction of the bubble in to the chamber filled with BAL. Furthermore, for 5 quasi static cycling the bubble was first reduced and then enlarged in a stepwise fashion between 0.5 and 2.5 atm. During the experiment, the chamber was kept at 37°C. Bubble volume, area and surface tension (*γ*) were calculated from digitized bubble images using height and diameter [33].

### In-vitro toxicity tests

Lung epithelial cells (type II alveolar epithelial cell line A549, CCL-185, [34,35]) were grown in RPMI 1640 medium plus 10% fetal calf serum in cell culture flasks. Subcultures were grown in 96 well microtitre plates, washed two times with PBS and incubated in 100 μl PBS containing 0.1 – 55 mM (0.002 – 1.0%) NCT, 0.1 – 1 mM NCT plus 0.37 – 3.74 mM (0.02 – 0.002%) ammonium chloride for 30 min at 37°C and pH 7.4. Subsequently, cell viability was evaluated by trypan blue exclusion tests and by CellTiter 96 Aqueous One Solution Cell Proliferation Assay (Promega Corporation, Madison, USA) in separate experiments. In the cell proliferation assay, 20 μl MTS solution [3-(4,5-dimethylthiazol-2-yl)-5-(3-carboxymethoxyphenyl)-2-(4-sulfophenyl)-2H-tetrazolium] was added and the cells were incubated for further 3 h. Then the formed formazan was quantified spectrophotometrically at 490 nm according to the instructions of the manufacturer. Controls were performed with PBS without additives, 1% taurine, and 1% ammonium chloride.

### Statistical Analysis

For haemodynamics, blood gas and respiratory measurements a two-way analysis of variance was used to determine inter- and intragroup differences. Significant differences were *post-hoc *analyzed with the Newman-Keuls test. Data are presented as mean ± SD. One-way ANOVA and Dunnett's multiple comparison test were applied for in-vitro toxicity tests. Chi-square and Fisher's test were used for evaluation of changes found in histology. Descriptive statistics were applied for electron microscopy and surfactometry. Cytokines were evaluated by standard statistical nonparametric tests for group differences, and correlations were performed using the software package SPSS 13 for MacOSX. p < 0.05 was considered significant for all tests.

## Results

### Pulmonary gas exchange

The following parameters of pulmonary gas exchange turned out as the most sensitive ones to detect any impact of the inhaled test substances.

**Figure 1:**
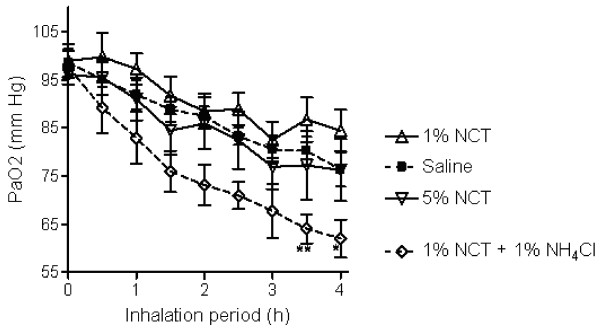
**Arterial partial pressure of oxygen (PaO_2_) of the pigs during the inhalation period**. Mean values ± SEM of 6–7 animals. P = 0.014 between the control and 1% NCT + 1% NH_4_Cl, p > 0.05 between all other groups. * p < 0.05 for single values; ** p < 0.01 for single values compared to the control.

Arterial partial pressure of oxygen (PaO_2_) values at baseline were within normal range in all animals without any intergroup difference (97 mmHg ± 7.6), reflecting healthy, anesthetized and ventilated animals. PaO_2 _decreased significantly over the observation period of 4 hours in all animals (Figure 1). No difference in PaO_2 _to controls was seen in those animals receiving 1% NCT or 5% NCT, whereas inhalation with 1% NCT + 1% NH_4_Cl led to significantly lower PaO_2 _values (62 ± 9.6 mmHg vs. 76 ± 9.2 mmHg, p = 0.014 after 4 h).

**Figure 2:**
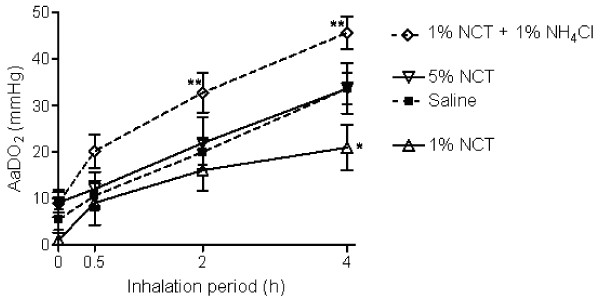
**Alveolo-arterial difference of oxygen partial pressure (AaDO2) of the pigs during the inhalation period**. Mean values ± SEM of 6–7 animals. p < 0.01 for 1% NCT + 1% NH_4_Cl compared to saline and 1% NCT; p < 0.05 (0.016) between saline and 1% NCT. * p < 0.05 for single values; ** p < 0.01 for single values compared to the control.

Alveolo-arterial difference of oxygen partial pressure (AaDO_2_) correspondingly increased in all animals during the experimental periode and was highest in the NCT + NH_4_Cl group (9.0 ± 7.2 at the baseline to 45.7 ± 8.73 after 4 h, p = 0.00014), Figure 2. After 4 hours, AaDO_2 _was significantly higher in the NCT + NH_4_Cl group (p = 0.004), but not different from controls in the 5% NCT group. In sharp contrast, AaDO_2 _was even lower in the 1% NCT group (21.0 ± 13.09 vs. 33.7 ± 9.03, p = 0.016). Mixed venous PO_2 _did not differ in the animals (37 ± 2.9 mmHg) and no change was observed during the experimental period. PaCO_2 _levels stayed within normal range (37 ± 2.8 mmHg), entirely without any inter- or intragroup changes.

The pressure of mechanical ventilation was started with mean values between 23.2 and 23.7 mmHg in all groups. To control for physiological end-tidal CO_2_-levels at 4 h, a positive inspiratory pressure of 30.0 ± 5.2 was necessary for the control animals, 30.3 ± 7.5 for 1% NCT, 32.8 ± 6.4 for 5% NCT, and 34.2 ± 4.8 for NCT + NH_4_Cl animals, respectively. The differences were not significant (p > 0.1), though the values might also indicate a smaller effect of 1% NCT on gas exchange than 5% NCT and the combination. Ventilation-perfusion distributions determined with MIGET showed no differences between the test (normal VA/Q of Q ratio after 4 hours 97% for 1% NCT, 95% for 5% NCT) and control groups (96%), except for NCT + NH4Cl (86%, p < 0.05).

### Haemodynamics

**Figure 3:**
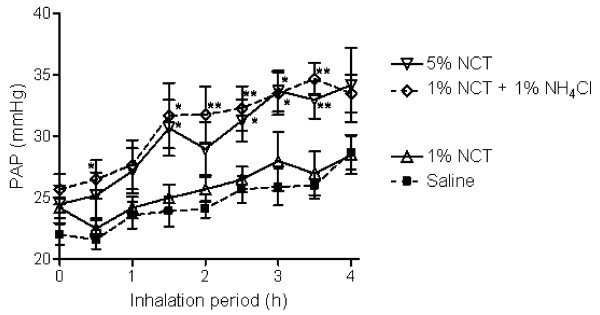
**Pulmonary artery pressure (PAP) of the pigs during the inhalation period**. Mean values ± SEM of 6–7 animals. p < 0.01 for 1% NCT + 1% NH_4_Cl and 5% NCT compared to saline and 1% NCT; p > 0.05 between saline and 1% NCT. * p < 0.05 for single values; ** p < 0.01 for single values compared to the control.

Pulmonary artery pressure as a further highly sensitive parameter for functional pulmonary disorders increased over the observation period in all animals (from 22.0 ± 2.2 mmHg to 28.7 ± 3.6 mmHg in controls). 1% NCT showed the smallest increase of PAP without difference to saline (p = 0.9), while it was higher after 4 hours with 5% NCT (p = 0.02) and NCT + NH_4_Cl (p = 0.05) (Figure 3).

Systemic arterial pressure remained constant within physiological range over the entire experimental period in all animals alike without any inter-or intragroup differences.

Heart rate at the 4 hours measurement was not significantly different from controls in both NCT groups, whereas it significantly increased by NCT + NH_4_Cl (104 ± 5.4/min to 136 ± 16.9/min, p = 0.00002), resulting in a significant difference to controls after 4 hours (111 ± 21.3/min, p = 0.00036).

### Pharmacokinetics

The taurine levels in serum ranged between 110 and 186 μM (0 h), and 85 and 172 μM (4 h), and they did not increase in any of the test groups and in the control group. However, the cystine levels increased significantly in the pigs treated with 5% NCT from 35.5 ± 7.9 μM (0 h) to 52.7 ± 4.2 μM (2 h) and 51.9 ± 6.2 μM (4 h) (p < 0.01), while they remained constant in all other animals. No changes were observed in further 27 amino acids compared to the controls.

We could not detect oxidation capacity in bronchial lavage performed immediately after euthanizing the animals. Therefore, local inactivation of NCT below detectable levels took place within 30 min.

### Histology

Histological investigations revealed signs for inflammatory reactions in all groups. However, there were no statistical differences between the test groups and the control group. Results are detailed in Table 1. In two animals treated with 5% NCT detached epithelial cells were found, which was not the case in the other groups (p = 0.065).

**Table 1:** Number of animals with histological changes in the lung subsequent to the inhalations

	**NaCl** **(n = 7)**	**1% NCT** **(n = 7)**	**5% NCT** **(n = 6)**	**1% NCT + 1% NH_4_Cl** **(n = 6)**
*HE*				
Infiltration with	4	1	3	2
Neutrophilic granulocytes	4	5	5	3
Eosinophilic granulocytes	2	2	1	0
Lymphocytes	4	6	5	3
Erythrocytes	3	5	2	3
Atelectasis	3	2	1	3
Bronchiolitis	3	2	4	4
Bronchitis	6	2 *	2^†^	4
Lymphangiectasia	1	2	1	1
Capillary congestion	1	2	1	2
				
*PAS*				
Goblet cells increased	4	5	2	2
Increased production of mucus	4	5	2	3
Detached epithelial cells	0	0	2^‡^	0
Epithelial changes	0	0	1	0
				
*Elastica*				
Fragmentation of lamina elastica	3	1	2	3
Fragmentation of lamina externa	1	0	0	0
				
*Reticulin*				
Fibers collapsed	4	3	3	1
Thinned alveolar septa	3	2	2	3
Thickened alveolar septa	3	1	1	0
Emphysema	3	4	2	2
Bullae	1	2	1	2
Thickened pleura	1	0	0	0

* P = 0.051 versus control

† P = 0.08 versus control

‡ P = 0.065 versus control

P > 0.1 for all other frequencies versus control

### Electron microscopy

There were no ultrastructural changes of any cell type in transmission electron microscopy. Apart of a focal decrease of cristae, mitochondria were unremarkable in all groups, no lysosomal activation was also seen. Moreover, we found no differences in the thickness of the basal membranes in all groups examined. The thickness of the basal membrane showed an overall range of 95 ± 2 nm and the variability of the membrane thickness was comparable in all groups with values between 92 and 99 nm. In particular, in the 1% NCT group the basal membrane thickness was 95 ± 2 nm, in the 5% NCT group 93 ± 2.6 nm, in the 1% NCT plus 1% NH_4_Cl group 96 ± 2.8 nm, and in the saline group was 94 ± 2 nm. No clear evidence of hyperplasia of pneumocyte type II was found in any group.

### In situ determination of cytokines

From each animal two samples were analyzed, biopsies near the carina and from peripheral lung tissue. In 12 out of the 56 samples no sufficient mRNA could be isolated, most likely due to mRNA degradation in the sample. The adjusted transcript values (number/μl cDNA) are listed in Table 2. The expression of the investigated genes was highly variable within each group and might reflect the variable degree of inflammation observed in the histological examination. This view is supported by a significant correlation between the inflammatory markers within the animals (with exception of the relation between HO-1 and TNFalpha) and the lack of significant differences between the investigated groups (p > 0.1 versus control in all cases). There was also a significant correlation in the expression of HO-1, IL-1beta and IL-8 gene expression between the samples taken near the carina and from peripheral.

**Table 2:** Gene expression by qRT-PCR in lung samples after inhalation, adjusted mRNA transcript values (number/μl cDNA)^a^

		**1% NCT**	**5% NCT**	**NCT + NH_4_Cl**	**NaCl**
HO-1	near carina	2656 (1878)	1772 (1282)	3200 (3018)	1469 (1737)
	peripheral	5003 (2608)	2833 (2639)	4300 (3416)	2044 (1717)
TNF alpha	near carina	394.8 (157)	370.8 (190)	635.6 (440)	400.7 (198)
	peripheral	214.6 (79)	185.2 (72)	243.0 (286)	344.6 (316)
IL-1-beta	near carina	82.0 (104)	116.5 (87)	73.4 (39)	75.0 (139)
	peripheral	77.8 (62)	58.3 (71)	247.6 (403)	164.0 (266)
IL-8	near carina	469.6 (395)	704.5 (991)	191.2 (93)	274.0 (475)
	peripheral	846.2 (506)	772.5 (895)	1426.2 (1670)	779.0 (1187)

^a ^mean values and (SD)

### Surfactant

**Figure 4:**
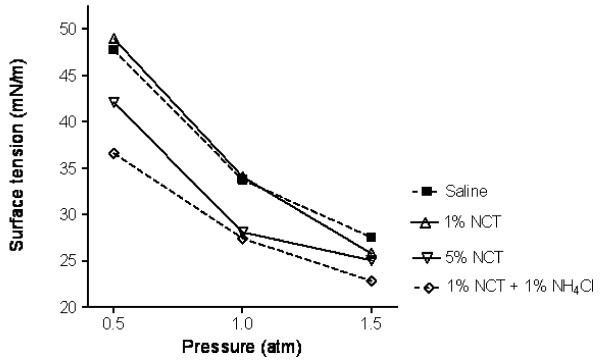
**Surface tension of bronchial lavage samples under different pressure measured by captive bubble surfactometry**. Mean values of two samples from pigs treated with NCT+NH_4_Cl and 0.9% NaCl, respectively. Single values from one pig treated with 1% NCT and 5% NCT, respectively.

The surface tension under different pressure at the end of the fifth quasi-static compression was similar in the control and test groups (Figure 4). The inclination of the curves from control and test samples was equal (Figure 4). NCT did not impact the function of surfactant. Minimal surface tension values were relatively high, probably because of the inflammation found with histology (see above).

### In-vitro toxicity tests

**Figure 5:**
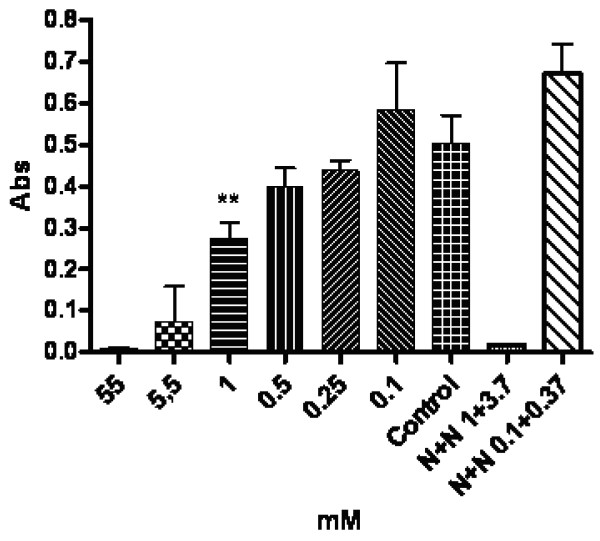
**Mitochondrial metabolism of A549 lung epithelial cells after 30 min incubation in NCT or NCTplus NH_4_Cl (N+N) at different concentrations in PBS in vitro, evaluated by the MTS test**. Mean values ± SEM of five independent experiments. ** p < 0.01 for this and lower values compared to the control (PBS without additives). Abs absorption at 490 nm.

Lung epithelial cells (A549) tolerated 0.25 mM NCT for 30 min. There was > 95% viability in the trypan blue exclusion test and no difference to the controls in the MTS test. NCT (0.5 mM) caused a slight but significant decrease of cell metabolism, while > 95% of the cells were still viable by trypan exclusion. In the latter test, 50% of the cells were killed by 1 mM, 83% (range 70–100%) by 11 mM, and > 95% by 55 mM NCT (mean values of 3 independent experiments). The corresponding loss of metabolism is depicted in Figure 5.

Chloramine T, another active chlorine compound used in human medicine, was investigated in parallel for comparison using the trypan blue exclusion test. The tolerated concentration of chloramine T, i.e. 0.01 mM, was 25-fold lower than that of NCT. Higher concentrations of chloramine T led to 10–30% (0.1 mM), 50–100% (0.5 mM), and > 95% loss of viability (1 mM) (range of 3 independent experiments).

## Discussion

Being a mild endogenous oxidant, NCT proved to be an antiseptic suitable for application to both the skin and mucous membranes of different body cavities such as paranasal sinuses [13,14,36,37]. The substance seems to be particularly applicable to tissues for which other antiseptics are avoided because of toxic side effects. Such a field is inhalative application. For special indications aerosolized antibiotics and antifungals have been used successfully, e.g. for treatment or prophylaxis of Pneumocystis jirovecii, Pseudomonas aeruginosa, and fungal infections [17,18,38]. Because of some uncertainties regarding efficacy and toxicity of the presently available therapeutics, the development of improved antimicrobial agents for inhalation seems to be suggestive. Antiseptics may be of advantage because of their broad-spectrum activity and largely the absence of antimicrobial resistance. Toxicity is a major point of concern with these compounds when administered to body cavities, but NCT seems to be well tolerated in the bronchopulmonary system according to our results.

Lung epithelial cells (A549) tolerated about 0.5 mM NCT and were similarly susceptible as other body cells [13,39], e.g. macrophages and keratinocytes. Moreover, our findings are in accordance with a previous report where taurine protected lung epithelial cells from HOCl-induced injury by conversion of HOCl to the low toxic NCT [20]. It is very important to mention that the clinically applied NCT concentration tolerated by different tissues is about 100-fold higher (55 mM) than that of monolayers in the cell culture [13,20,39]. Therefore, cell culture systems are useful for comparison of antiseptics (e.g. chloramine T and NCT, [13]) and to predict to a certain extent in vivo toxicity, as it was the case with NCT plus ammonium in our study. However, it is finally decisive that a clinically effective dose is tolerated in both animal models and in man. As expected and similar to keratinocytes [13], the stronger oxidant chloramine T damaged lung epithelial cells much more than NCT.

We chose the pig model since it has high similarity to human, ventilation and circulation parameters can be monitored, inhalations can be performed in a similar way, and sufficient amounts of serum and tissue can be removed for evaluation. A limitation in our unit was that the experimentation in each animal had to be performed within one day, so that dosing and monitoring for longer periods was not possible. This disadvantage may have been compensated for to a certain degree by the performed intensive treatment schedule.

In accordance with our previous clinical studies in both human beings and in animals (e.g. [12-14,36,37,40-42], the concentration of 1% NCT (55 mM) seems to be very well tolerated also in the bronchopulmonary system. Also the inhalative way has to be regarded as a topical one since the amino acid concentrations in the blood, particularly of taurine, did not change in all groups. This was the case despite the high concentration gradient of taurine applied locally (5% NCT equals 275 mM and 400 mM taurine after reduction of the active chlorine) and in the serum of the pigs (85–186 μM). The only hint for a small, negligible resorption of high inhaled concentrations could have been the increase in cystine in the 5% NCT group. A theoretic explanation for that is oxidation of cysteine to cystine, which takes place in the presence of NCT. It may be that a minimal increase of taurine after application of 5% NCT could not be detected within the standard deviation. On the other hand, the blood level of methionine remained constant, although it is oxidized by NCT, too. Presently it can be stated that 1% NCT ± 1% NH_4_Cl does not reach the circulation in detectable amounts, while minimal amounts are possible with 5% NCT.

Since the reaction with thio groups leads to immediate inactivation of NCT, the oxidative activity decreased quickly and could not be measured in situ approximately 30 min after the last dosing. The short decomposition time in vivo is in accordance with previous studies in the eye (5–15 min, [36,43]). Longer times of a few hours are only possible if a fluid level containing NCT is kept in body cavities [37].

No tested clinical and laboratory parameter in the 1% NCT group worsened more than in the saline group during the experimental period. With inhalation of chloramine T, a markedly stronger active chlorine compound than NCT, bronchopulmonary irritation and allergic sensitization with asthmatic symptoms are known [44]. The lower reactivity of NCT and the fact that it deals with an endogenous amino acid derivative are obviously decisive for its improved tolerability. If the concentration was increased to 5% in our pig model, only one parameter (pulmonary artery pressure) worsened significantly compared to placebo. A similarly high safety of NCT could be found for instance in the eye [36], in the ear [45] and in the cow udder [40], where concentrations of 3–10% caused local irritation, too, but no severe or long-term side effects. Allergic reactions against NCT seem improbable since it is produced upon every inflammation by leukocytes and it is a small molecule (amino acid derivative), and such reactions have not yet been observed in clinical studies.

As a more potent chloramine formulation and as a positive control, we chose a 1% combination of NCT and ammonium chloride (NH_4_Cl). This leads to formation of about 0.025% (4.9 mM) monochloramine (NH_2_Cl) which is only little more reactive but significantly more lipophilic than the hydrophilic NCT [46]. The consequence is increased penetration of pathogens but also body cells and tissue, which may be wanted in some indications, e.g. in the eye for treatment of viral conjunctivitis [11,43]. The concentration of 1% of both constituents is very high and cannot be applied to the eye, but a tenfold dilution is very well tolerated [43]. As expected, in the pig model oxygenation and perfusion parameters changed after inhalation with 1% NCT + 1% NH_4_Cl compared to saline and NCT. Since systemic absorption of NCT ± NH_4_Cl was not detected and low amounts in the blood would be inactivated immediately, these changes must have been caused by direct impact on lung epithelial and endothelial cells. We expected a thickening of basal membranes, but there were no changes at all found by electron microscopy. Therefore, functional abnormalities of the lung cells must be assumed which did not lead to structural changes. In vitro, lung epithelial cells demonstrated about tenfold higher sensitivity to NCT + NH_4_Cl than to NCT, which is in accordance to the in vivo results, at which the threshold tolerated concentration of the combination remains to be tested in vivo.

Histologic evaluation did not reveal an explanation for the effects of 1% NCT + 1% NH_4_Cl and 5% NCT, too. The abnormalities found, mainly infiltration with leukocytes, and local atelectasis and emphysema, are well known to occur during the anaesthesia of pigs [47]. It cannot be excluded that these changes might have overlapped and masked minimal ones caused by the test medication. In any case the absence of a significant impact clearly visible in histology indicates the relatively high tolerance of NCT with and without NH_4_Cl by tissue.

Furthermore, the function of surfactant seems not to be significantly influenced by the medication. It is true that the test procedure using the captive bubble surfactometer is very complicated, laborious, and affected with high deviations, and it was possible to perform only a few tests in the present study. Therefore, small differences between the tested groups cannot be excluded, but a massive destruction of surfactant or its producing cells did not take place. This would have caused much more intensive clinical problems. In accordance with that, NCT as a mild active chlorine compound mainly reacts with thio and amino groups [6] and has no targets with the phospholipid constituents of surfactant which are important for its function [48].

The fact that one oxygenation parameter (AaDO_2_) was even significantly better at the end of the applications in the pigs treated with 1% NCT compared to saline may be regarded as pure chance. On the other hand, anti-inflammatory effects might be caused by NCT since in its presence proinflammatory cytokines and interleukins have been shown to be produced in lower amounts by stimulated leukocytes in vitro [4,5,16]. Some effects in part contrasting these results have also been found, i.e. significant induction of IL-1beta, IL-8, TNF-alpha and HO-1 gene expression in human PBMC (Giese T, Heidelberg, unpublished results). When NCT was applied to treat infections in our clinical studies, specific investigations to quantify a possible anti-inflammatory effect and to differentiate it from the antimicrobial one have not been performed. Some signs for clinically relevant anti-inflammatory effects may be derived from phase 2a studies on chronic rhinosinusitis and, above all, postoperative ear care after tympanoplasty, where deswelling and drying effects were found in the absence of infection [37,41]. In the present study we tried to address this question specifically for the first time in vivo by measurements of IL-1beta, IL-8, TNF-alpha and HO-1 gene expression. However, no conclusive results could be gained from lung tissue in vivo. As observed during histo-pathological examination, the experimental procedure triggered a variable degree of injury-associated inflammation in the lung tissue. This reaction might have overridden the more subtle changes caused by NCT and NCT+NH_4_Cl. However, this hypothesis could not be proven in the experiment due the limited number of investigated samples.

Summing up, inhaled NCT at a concentration of 1% was well tolerated in the pig model. Further investigations of its tolerability in human beings and on its possible efficacy in the infected bronchopulmonary system are justified.

## Conclusion

The antiseptic NCT was well tolerated at a concentration of 1% upon inhalation in the pig model, while 5% demonstrated small effects on pulmonary artery pressure, and 1% NCT + 1% NH_4_Cl small effects on gas exchange and haemodynamics. Therefore, 1% NCT may be suitable for inhalative application, while for the combination with ammonium a dose reduction is probably required.

## Competing interests

The authors declare that they have no competing interests.

## Authors' contributions

All authors read, performed according corrections, and approved the final manuscript. Additionally, the authors' tasks were specifically the following: RG, planning of the study, establishment of the inhalation devices, evaluation, writing of the manuscript; BT, performance and guidance of the animal work, evaluation, writing of the animal model; AP, animal work, sample preparation and collection, data management and evaluation; LB, performance and evaluation of histology; HP, HR, MP, MH, performance of the animal model including the inhalations, sample collection, data management; CW, performance of pharmacokinetic tests and surfactant studies and evaluation; HJS, performance and evaluation of electron microscopy; TG, in situ determination of cytokines and evaluation; AW, establishment of bubble surfactometry, guidance of surfactant studies and evaluation; SSB, guidance and evaluation of pharmacokinetic tests; WG, planning of the study, evaluation; RA, planning, evaluation; CS, guidance, performance and evaluation of histology and electron microscopy, writing of the histological part of the manuscript; MN, planning of the study, coordination of all involved scientists and of their work, evaluation, writing of the manuscript; AL, guidance and coordination of the animal tests, evaluation and writing of the animal model.

## Pre-publication history

The pre-publication history for this paper can be accessed here:


